# The Meaning of Lymphadenopathies During Adjuvant Durvalumab After Chemoradiotherapy for Lung Cancer: Thinking Beyond Disease Progression

**DOI:** 10.7759/cureus.26729

**Published:** 2022-07-11

**Authors:** Marcos Pantarotto, Rita Barata, Ricardo Coelho, Virginia Sousa, Catarina Carvalheiro, Ines Rolim, Patricia Garrido, Nuno GIl, Filipa Duarte-Ramos, Fernanda S Tonin

**Affiliations:** 1 Oncology, Champalimaud Foundation, Lisbon, PRT; 2 Lung Unit, Champalimaud Foundation, Lisbon, PRT; 3 Dermatology, Champalimaud Foundation, Lisbon, PRT; 4 Faculty of Pharmacy, University of Lisbon, Lisbon, PRT; 5 Health & Technology Research Center, Escola Superior de Tecnologia da Saúde de Lisboa, Instituto Politécnico de Lisboa, Lisbon, PRT

**Keywords:** immune-checkpoint inhibitors, pseudoprogression, immune-related adverse events, lung cancer, differential diagnosis

## Abstract

Immune-checkpoint inhibitors (ICIs) have become the mainstay of treatment for many malignancies. With this new strategy, relevant immune-related adverse events (irAEs) have been reported, some of which can be mistaken for disease progression.

To better illustrate the current challenges in diagnosing and managing a patient under adjuvant ICI treatment, we present the case of a 67-year-old female patient with stage IIIB unresectable, epidermal growth factor receptor (EGFR)-mutated, non-small-cell lung cancer who was initially treated with chemoradiotherapy, followed by immunotherapy with durvalumab. During the course of immunotherapy, the patient presented with madarosis and erythematous and endured skin lesions, in addition to lymphadenopathies and pulmonary infiltrates. She was started on first-line palliative treatment with an EGFR tyrosine kinase inhibitor. After reviewing the case, a multidisciplinary team meeting suggested diagnostic procedures, including a transbronchial needle aspiration from mediastinal lymph nodes. The histologic examination showed chronic systemic inflammation and non-caseating granulomas of the sarcoid type.

In this case, palliative treatment was suspended and systemic therapy with prednisolone was initiated. The patient became asymptomatic and the previously observed radiologic abnormalities resolved. This case highlights the importance of early recognition and appropriate treatment of irAEs, mainly because these conditions remain poorly understood and are probably underdiagnosed. Considering differential diagnosis is paramount to guide clinical management, despite curative or palliative treatment intent.

## Introduction

In the last few years, immune-checkpoint inhibitor (ICI) indications evolved from the palliative to the curative setting. Durvalumab, a high-affinity human immunoglobulin G1 monoclonal antibody that blocks the binding of programmed death-ligand 1 (PD-L1) on tumor cells or antigen-presenting cells with programmed cell death protein 1 (PD-1) and CD80, was approved in Europe in 2018 for consolidation after chemoradiotherapy for stage III, unresectable, non-small-cell lung cancer (NSCLC). Behind this fast transition from palliative treatment to earlier stages of the disease is a significantly increased overall survival, with a favorable side effect profile [1,2]. However uncommon, relevant immune-related adverse events (irAE), a unique spectrum of adverse reactions of ICIs that resemble autoimmune responses, can be challenging to diagnose and are mainly represented by conditions rarely observed in oncology practice. In addition, some irAEs may mimic disease progression in their clinical course, leading to treatment decisions that can be disadvantageous to the patient [3,4].

In this case report, we present the case of a female patient with advanced NSCLC treated with chemoradiotherapy followed by durvalumab where a multidisciplinary evaluation and a diagnostic procedure altered the diagnosis of disease progression.

## Case presentation

A 67-year-old female patient, a retired physician, non-smoker but with second-hand exposure to smoke, with an unremarkable medical history, presented to our clinic in December 2020 for a second opinion consultation. She had been diagnosed with stage IIIB (cT1b cN3 cM0, American Joint Committee on Cancer (AJCC) eighth edition) lepidic lung adenocarcinoma with epidermal growth factor receptor (EGFR) mutation (exon 19 deletion; PD-L1 = 1% via a transthoracic needle biopsy) in March 2019 after a routine chest X-ray revealed a suspicious nodule in the lung. A thoracic computer tomography (CT) scan confirmed a 20 mm nodule in the left lower lung lobe with no mediastinal adenopathies. However, a whole-body positron emission tomography-computed tomography (PET-CT) identified high fluorodeoxyglucose (FDG) uptake in the lung lesion and over the 4R mediastinal station, leading to the cN3 staging.

Between April and July 2019, she received concomitant chemotherapy with cisplatin and vinorelbine (four cycles) and radiation therapy to a total dose of 60 Gy in 33 sessions. In December 2019, despite a long interval since the end of chemoradiotherapy justified by administrative reasons, she received adjuvant immunotherapy with intravenous (IV) durvalumab 10 mg/kg every two weeks.

In November 2020, a follow-up thoracic CT scan showed mediastinal adenopathies, and an ^18^F-FDG PET-CT was ordered. In December 2020, one year after the introduction of durvalumab therapy, the PET-CT was performed and revealed lymphatic (mediastinal) and pulmonary disease progression. Her physician decided to start palliative treatment with the EGFR-tyrosine kinase inhibitor (TKI) gefitinib, following which the patient consulted us for a second opinion.

On our initial evaluation, the patient had a performance status (Zubrod) of 1, with the relevant findings of dry skin, one interscapular crusted skin lesion, and madarosis associated with erythematous, indurated skin nodules. The patient reported that the skin lesions and madarosis had appeared four months after the beginning of immunotherapy and had been stable since then. A thoracic CT scan showed bilateral pulmonary infiltrates with left predominance and bilateral hilar and mediastinal enlargement of the lymph nodes. The lung function tests (LFTs) revealed a moderate decrease in the diffusing capacity for carbon monoxide (DLCO) with average lung volumes. We opted to continue the EGFR-TKI while we obtained histologic confirmation of disease progression and evaluated the skin alterations.

**Figure 1:**
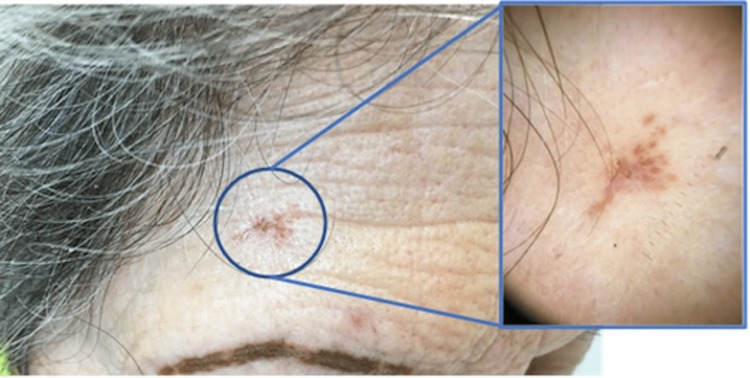
An aspect of the madarosis with micropigmentation of the eyebrows, along with a pericentimetric, orange-red right frontal lesion (magnified in the box).

**Figure 2:**
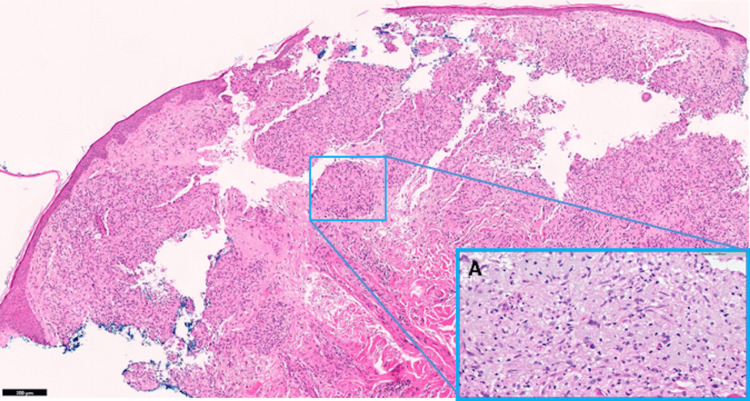
Skin biopsy showing (A) an area of granulomatous inflammation of superficial and deep dermis composed of well-formed granulomas with scattered multinucleated giant cells and absence of necrosis (hematoxylin and eosin).

During a dermatological evaluation, a reddish macula on the right frontal region was biopsied (Figure 1). The pathologic examination of this lesion revealed an exuberant chronic inflammatory granulomatous reaction involving both superficial and deep dermis constituted by non-caseating granulomas with multinucleated giant cells (Figure 2).

**Figure 3:**
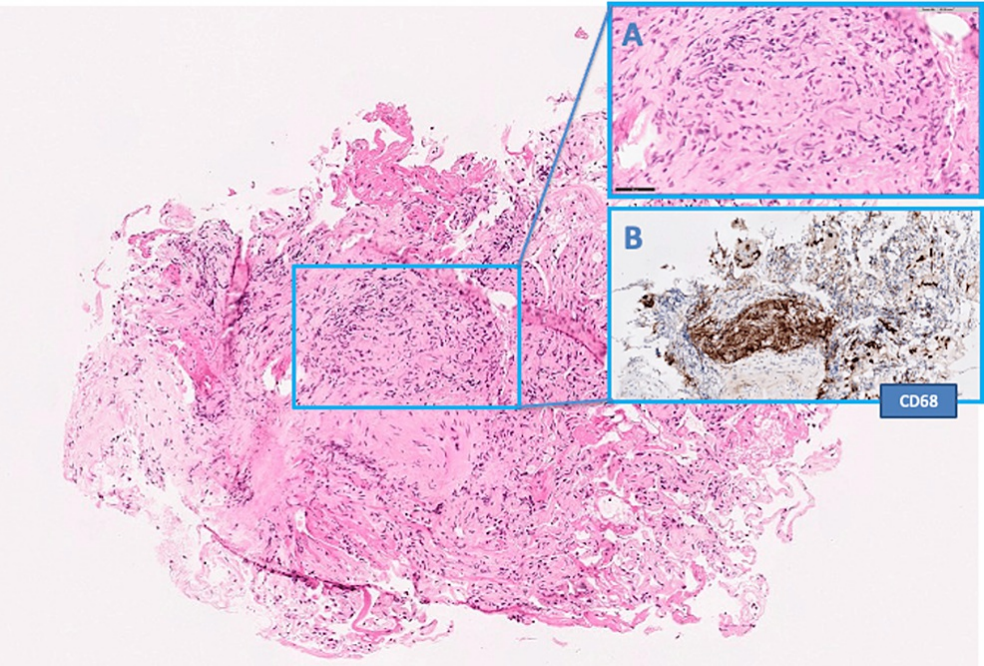
Lung biopsy showing (A) granulomatous inflammation with non-necrotizing (sarcoid-like) granulomas, highlighted with CD68 immunostaining for macrophages (B). There was no evidence of microorganisms with special stains (periodic acid-Schiff, Grocott, and Ziehl-Neelsen).

A multidisciplinary meeting discussion suggested a diagnostic procedure to evaluate for a systemic immunotherapy-related sarcoid-like reaction (SLR). Transbronchial lung biopsy and transbronchial needle aspiration (TBNA) from the lymph nodes were obtained via bronchoscopy and endobronchial ultrasound (EBUS), respectively, confirming chronic inflammation and non-caseating granulomas of the sarcoid type (Figure 3).

A diagnosis of durvalumab-associated systemic sarcoidosis was made with skin, lung, and lymph node involvement. Palliative treatment with EGFR-TKI was suspended in February 2021, and systemic therapy with prednisolone 0.5 mg/kg/d was initiated under specialized pulmonology consultation. After progressive tapering, we observed complete resolution of the radiologic pulmonary changes and adenopathies, normalizing DLCO in the LFTs and improving the patient’s complaints.

**Figure 4:**
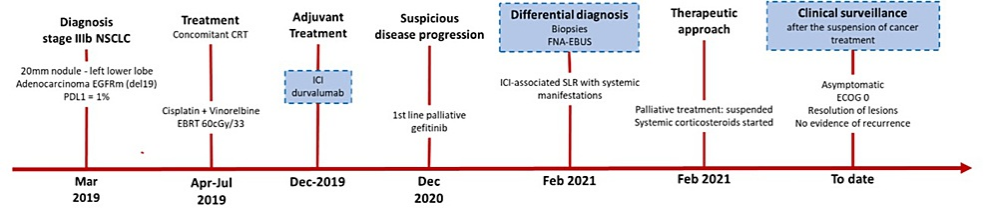
Timeline of the case report: main outcomes. NSCLC: non-small-cell lung cancer; EGFR: epidermal growth factor receptor; PDL1: programmed death-ligand 1; CRT: chemoradiotherapy; EBRT: external beam radiation therapy; ICI: immune-checkpoint inhibitor; FNA: fine-needle aspiration; EBUS: endobronchial ultrasound; SLR: sarcoid-like reaction; ECOG: Eastern Cooperative Oncology Group

As of February 2022, the patient was asymptomatic, and a thoracic CT scan demonstrated resolution of the adenopathies and no further pulmonary infiltrates, except for the fibrosis-related changes to the radiation treatment. The patient is alive with no evidence of disease progression to date and without oncologic treatment (see case timeline in Figure 4).

## Discussion

Sarcoidosis and SLRs are systemic inflammatory disorders of unknown etiology characterized by the development of non-caseating granulomas in various organs [5,6]. Although it can occur at any site, the most frequent clinical patterns of sarcoidosis consist of skin involvement and intrathoracic lymphadenopathy, usually mediastinal and hilar, with or without pulmonary parenchymal disease [5,7]. Mortality in SLR is mainly caused by respiratory failure due to pulmonary fibrosis, central nervous system involvement, and cardiac damage [6].

Since the late 1950s, there has been an ongoing debate on the relationship between SLRs and malignancies, given that sarcoidosis may precede, be diagnosed concurrently with, or follow an oncologic disease [5,6]. Recently, literature has indicated a common etiology in at least 25% of patients [8]. An analysis of both clinical and radiological features of a series of 29 granulomatosis patients with pre-existing cancer (mainly breast cancer and lymphoma) additionally showed that sarcoidosis following a malignancy is usually indistinguishable from idiopathic sarcoidosis, suggesting that the diagnosis and management of this condition should be further standardized regardless of the cause. Studies have also highlighted that some therapies (e.g., antiretroviral drugs, interferons, tumor necrosis factor antagonists) might lead to the development of SLRs [7]. A review of the World Health Organization (WHO) pharmacovigilance database in 2019 strongly associated 2,425 drug-induced sarcoidosis cases with some drug exposure, of which around 6% referred to cancer target therapies [9].

Immunotherapy targeting specific proteins (cytotoxic T-lymphocyte antigen 4 (CTLA-4), PD-1, PD-L1) comprises a relatively new class of treatments with proven efficacy for a broad spectrum of solid organ and hematologic malignancies [3]. Their mechanism of action leads to an endogenous immune response with cytotoxic T-cell activation and subsequent elimination of cancer cells. However, this immune-based response alteration can trigger intolerance and a new spectrum of irAEs [10]. In the past years, ICIs (e.g., pembrolizumab, nivolumab, atezolizumab, durvalumab, avelumab, ipilimumab) and BRAF/MEK inhibitors (e.g., vemurafenib, dabrafenib, combination with trametinib or cobimetinib) have been associated with a wide range of irAEs, but more commonly with skin toxicities, endocrinopathies (mainly thyroiditis), and pneumonitis [6,10]. There is no precise data about the frequency of SLRs in cases treated by ICIs, and it is usually considered a rare adverse event, with rates of 2-5% within melanoma clinical trials [5,10]. Conversely, Chorti et al. (2020) recently reported a disproportionally high rate (around 20%) of SLRs in a setting of melanoma patients receiving ICI as an adjuvant treatment [11].

The diagnosis and management of SLRs can be challenging for many reasons. Clinical presentation can vary from asymptomatic to very diverse (e.g., cough, dyspnea, skin lesions, arthralgia) [12,13] and can be associated with various drugs [14]. Moreover, immune-related pulmonary reactions may mimic disease progression or metastasis on imaging and examination, leading to an urge to pursue oncologic treatment. Finally, as biopsies for histological confirmation of sarcoidosis and disease staging are not systematically performed, SLRs can be underestimated as a confounding diagnosis [3]. The time between starting ICI and the identification of SLRs is also poorly described and ranges widely, with reports ranging from three weeks to over two years [7,13].

Moreover, the management of SLRs is highly controversial, and no clear guidance exists. Although it usually improves upon ICI discontinuation (with or without corticosteroids), some granulomatous reactions and asymptomatic cases resolve without treatment [3,10]. According to an analysis of the ImmunoCancer International Registry (18 countries; n =32 patients with biopsy-proven sarcoidosis), ICI-related SLR seems to have a benign outcome most often; continuing or resuming the therapy seems to be safe for oncologists [15].

Finally, despite melanoma being the most common underlying malignancy, ICI-associated SLR has also been reported in renal, urothelial, and breast cancers, mainly with the use of ipilimumab, nivolumab, pembrolizumab, or ICI combinations [5,6,10]. Reports of SLR in lung cancer also increased in the past years, with an estimated incidence of around 3% [15]. This may be justified, among others, due to the widespread use of ICIs in this scenario, as their recent introduction significantly modified the therapeutic algorithm of this cancer, with significant improvements in patients’ clinical results and health-related quality of life [16-19].

This case warns of some autoimmune conditions that may act as confounding factors of neoplastic disease progression or pseudoprogression during and after immune-directed treatments. The recognition and appropriate treatment of autoimmune complications are crucial to avoid hindrance to overall survival and quality of life.

Although the mechanism of sarcoidosis is not fully understood, ICI‐induced SLR (including inhibitory receptor PD-1 and its ligand PD-L1) may be related to the modulation of T-lymphocytes and antigens derived from dead tumor cells (PD-1+ CD4+T), with a median estimated onset of the adverse event of around 14 weeks after ICI initiation [19,20]. No apparent ICI dose threshold for developing irAE exists [3]. Additionally, as the histopathology from reported cases of ICI-induced SLR is similar to idiopathic sarcoidosis (e.g., biopsy specimens reveal focal infiltration by non-caseating epithelioid and giant cell granulomas), the role of differential diagnosis is paramount [7,11].

SLR diagnosis is challenging as it cannot be established unless alternative causes for sarcoidosis (e.g., inflammatory diseases, infections) have been excluded. In suspected SLR during cancer treatment, differentiation from malignancy progression is critical because it affects the decision‐making of whether the treatment should continue. This situation is especially complex in lung cancer patients as the enlargement of mediastinal and hilar nodes is often related to disease progression [13]; it can also be linked to inflammatory and infectious processes.

Conversely, mediastinal adenopathies do not secure the diagnosis of SLR and may represent reactive lymphadenopathy associated with an irAE [5,20]. A single-center retrospective analysis showed that patients with ICI-related SLR presented bilateral hilar lymphadenopathy and granulomatous reactions in lymph node biopsies [13]. In our patient, the co-existence of cutaneous lesions was helpful because they were easily accessible for biopsy, allowing a rapid and safe histological confirmation of granulomas. A case-control study performed in France (2016-2020), with 28 oncologic patients presenting SLR associated with immunotherapy, showed that histopathological results obtained from biopsies of different tissues (mediastinal lymph node, kidney, skin, liver, bronchial, lung, subdiaphragmatic lymph node) are sufficient to enable proper SLR diagnosis [12].

While a biopsy is considered the standard for investigating SLR, it is not always possible to pursue adequate tissue sampling. A few factors can make the process of biopsy challenging, such as the patient’s clinical status, the availability of appropriate technology and specialized technicians to make the biopsy, or the location of the lesions for biopsy. This is especially true for lung cancer as the tumors may not be readily accessible. Moreover, even when needed, repeated biopsies may not be feasible [21,22].

Literature on ICI-associated SLR remains scarce, and the severity of granulomas is usually unreported, which hampers further investigations. This can be due, among others, to the lack of standardization of the condition by some well-known systems and descriptive terminology criteria such as the US National Cancer Institute Common Terminology Criteria for Adverse Events (NCI-CTCAE). Nonetheless, the Society of Immunotherapy of Cancer (SITC) proposed a classification system for SLR [4]. In stage I, patients have localized SLR, while in stages II and above, patients present with extensive disease, extrapulmonary disease involving critical organ systems (e.g., myocardial, neurological, ocular, renal), or sarcoid-related hypercalcemia. Sarcoidosis cases in oncologic patients are mainly mild to moderate (grade I and II according to CTCAE) (around 93% of cases), with few cases judged as severe [12]. It was also observed that patients with SLR appear to have an improvement in overall survival (median not reached in the SLG cohort compared to 40.4 months in the control cohort (hazard ratio = 0.232, 95% confidence interval = 0.086-0.630; p = 0.0002), and no patient died because of sarcoidosis [12]. This evidence and other previous studies suggest that many irAEs, including SLRs, can predict a favorable prognosis with ICI use [5].

Treatment management of SLR is still highly controversial, and no specific guidelines exist. No prospective studies have focused on managing SLR as an irAE of ICI, and current recommendations are mainly based on clinical experience or real-life reports [13]. The most reliable way to confirm drug-derived toxicity is to discontinue and re-challenge the therapy to check for sequential improvement and recurrence of the adverse event. However, this approach may not be appropriate for life-saving drugs such as ICIs [3,5]. According to the Society for Immunotherapy of Cancer (SIC) Toxicity Management Working Group, asymptomatic cases of SLR do not need treatment. In case of severe symptoms (Table 1), holding ICI treatment and adding systemic corticosteroid therapy at the equivalent dose of prednisolone 1 mg/kg/day is recommended. Corticosteroid tapering and withdrawal must rely on the clinical response [3].

**Table 1:** Severity criteria of SLR according to SIC - Toxicity Management Working Group. DLCO: diffusing capacity for carbon monoxide; TLC: total lung capacity; FVC: forced vital capacity; SLR: sarcoid-like reaction; SIC: Society for Immunotherapy of Cancer

Severity criteria for sarcoid-like reactions
DLCO decrease of >20%
TLC decrease of >10%
FVC decrease of >15%
Persistent SLR symptoms
Radiological progression
Involvement of extrapulmonary organ systems
Hypercalcemia not otherwise explained

Although most cases (>60%) do not need systemic corticosteroids, the literature currently suggests that a significant proportion of patients finally discontinue immunotherapy due to the development of SLRs (around 50-60% of cases), primarily aiming at decreasing the severity of reactions [5,10]. Nonetheless, the analysis of the WHO database showed that only around 20% of granulomatous reactions improved with the reduction or suspension of the suspected therapy [9].

The decision to discontinue ICI may have a dual origin. First, because SLR can be interpreted as a threatening situation that needs to resolve, the safest way is to remove the trigger (i.e., ICI). Second, considering that the efficacy of immunotherapy may be impeded by the addition of systemic steroids to control SLR symptoms, many physicians prefer to hold the ICI [11]. Finally, avoiding the unnecessary use of immunosuppressive drugs in patients with irAEs is highly recommended [8,11]. It is also essential to consider the potential consequences of stopping anticancer treatment, particularly in the absence of clear management recommendations [4,13].

In our case, the patient was successfully treated with durvalumab, with no reported disease recurrence to date and resolution of SLR after systemic corticosteroids. Previous studies have also shown partial therapeutic response, stable disease, or complete remission of cancer in over 70% of patients who developed SLR lesions associated with ICI treatment over a median follow-up of around 12 months since treatment initiation [18,23,24]. Thus, active surveillance and differential diagnosis of SLR during the oncologic disease course are paramount to avoid unnecessary treatments (i.e., the continuation of palliative treatment) at the expense of better therapeutic approaches.

This study has some limitations. Although a systematic literature review would be methodologically more robust to cover the topic of ICI-associated SLR in lung cancer, we demonstrated through a narrative review that the literature is still scarce. Our discussion is grounded on the results of a case reported in our center; however, other cases may have slightly different outcomes and, thus, should be interpreted within their clinical contexts.

## Conclusions

Mediastinal lymphadenopathies during the course of immunotherapy should not be considered disease progression without a thorough patient examination and histologic evaluation. Sarcoidosis and SLR associated with ICI treatment remain an infrequent and poorly understood irAE in advanced NSCLC, probably underdiagnosed as biopsies for histological confirmation and grading are not systematically performed. Awareness of the potential associations between these conditions may alter clinical management, whether curative or palliative. This highlights the role of well-performed differential diagnosis from cancer progression as SLR manifestations may clinically and radiographically mimic disease recurrence. Protocols for the appropriate evaluation and management of these conditions in patients treated with ICI should be developed.
