# Correction: The Meaning of Lymphadenopathies During Adjuvant Durvalumab After Chemoradiotherapy for Lung Cancer: Thinking Beyond Disease Progression

**DOI:** 10.7759/cureus.c101

**Published:** 2023-02-13

**Authors:** Marcos Pantarotto, Rita Barata, Ricardo Coelho, Virginia Sousa, Catarina Carvalheiro, Ines Rolim, Patricia Garrido, Nuno GIl, Filipa Duarte-Ramos, Fernanda S Tonin

**Affiliations:** 1 Oncology, Champalimaud Foundation, Lisbon, PRT; 2 Lung Unit, Champalimaud Foundation, Lisbon, PRT; 3 Dermatology, Champalimaud Foundation, Lisbon, PRT; 4 Faculty of Pharmacy, University of Lisbon, Lisbon, PRT; 5 Health & Technology Research Center, Escola Superior de Tecnologia da Saúde de Lisboa, Instituto Politécnico de Lisboa, Lisbon, PRT

This article has been corrected at the request of the authors to include Virginia Sousa as fourth author as she was erroneously omitted during the submission process. The authors deeply regret that this error was not identified and addressed prior to publication.

 

